# High-Precision Marine Radar Object Detection Using Tiled Training and SAHI Enhanced YOLOv11-OBB

**DOI:** 10.3390/s26030942

**Published:** 2026-02-02

**Authors:** Sercan Külcü

**Affiliations:** Computer Engineering Department, Giresun University, Giresun 28200, Turkey; sercan.kulcu@giresun.edu.tr

**Keywords:** marine radar, object detection, oriented bounding boxes, SAHI, tiled training, deep learning, instance segmentation, small target detection

## Abstract

**Highlights:**

**What are the main findings?**

- The combination of tiled training, SAHI, and YOLOv11-OBB achieves over 0.95 mAP@0.5 on the challenging real-world DAAN marine radar dataset, outperforming standard full-image detection approaches.
- Oriented bounding boxes provide better localization and tighter fits for vessels compared to axis-aligned boxes and segmentation masks.

**What are the implications of the main findings?**

- High-precision detection with lightweight models enables real-time deployment on edge hardware suitable for small to medium-sized vessels.
- Accurate orientation and centroid estimation improve maritime tasks such as multi-target tracking and collision avoidance in cluttered environments.

**Abstract:**

Reliable object detection in marine radar imagery is critical for maritime situational awareness, collision avoidance, and autonomous navigation. However, it remains challenging due to sea clutter, small targets, and interference from fixed navigational aids. This study proposes a high-precision detection pipeline that integrates tiled training, Sliced Aided Hyper Inference (SAHI), and an oriented bounding box (OBB) variant of the lightweight YOLOv11 architecture. The proposed approach effectively addresses scale variability in Plan Position Indicator (PPI) radar images. Experiments were conducted on the real-world DAAN dataset provided by the German Aerospace Center (DLR). The dataset consists of 760 full-resolution radar frames containing multiple moving vessels, dynamic own-ship, and clutter sources. A semi-automatic contour-based annotation pipeline was developed to generate multi-format labels, including axis-aligned bounding boxes, oriented bounding boxes (OBBs), and instance segmentation masks, directly from radar echo characteristics. The results demonstrate that the tiled YOLOv11n-OBB model with SAHI achieves an mAP@0.5 exceeding 0.95, with a mean center localization error below 10 pixels. The proposed method shows better performance on small targets compared to standard full-image baselines and other YOLOv11 variants. Moreover, the lightweight models enable near real-time inference at 4–6 FPS on edge hardware. These findings indicate that OBBs and scale-aware strategies enhance detection precision in complex marine radar environments, providing practical advantages for tracking and navigation tasks.

## 1. Introduction

X-band marine radar is a critical sensor for maritime situational awareness, providing detection capabilities in various weather conditions, although its performance can be degraded in heavy rain due to signal attenuation and clutter [1]. However, reliable object detection remains challenging due to signal interference and the small size of distant targets. Traditional approaches predominantly relied on statistical signal processing. Zhou et al. proposed a Constant False Alarm Rate (CFAR) method based on superpixel segmentation to handle dynamic sea clutter distribution. Authors achieved a detection rate of 84.82% on the evaluated dataset [2]. Similarly, Li and Wang analyzed the Generalized Symbol (GS) detector, a nonparametric CFAR approach, demonstrating its robustness in varying sea states compared to parametric models [3]. To address unintentional interference between co-sailing radars, Mao et al. developed an anti-interference imaging framework using nonuniform sampling and dimension-reduction iterative adaptive approaches [4]. Furthermore, Wen et al. introduced a spatio-temporal domain joint filtering method based on 3D-FFT, which utilizes the linear wave dispersion relation to suppress clutter and improve signal-to-noise ratio [5].

Despite these advances, deep learning methods have outperformed classical techniques in complex marine environments. Early implementations by Chen et al. introduced Radar-PPInet, a YOLOv3-based network that showed higher detection probability over classical CFAR and Faster R-CNN models [6]. Chen et al. also developed Radar-YOLONet, a single-stage detector using CSPDarknet53 backbone and Power Non-Maximum Suppression (P-NMS) method for scanning radar images [7]. Focusing on low-resolution radar data, Chen et al. proposed Marine-Faster R-CNN model, a two-stage detection framework enhanced with a Feature Fusion Network (FFN) to improve robustness against noise and enhance the detection of small targets. Their approach reported a recall of 93.65% [8]. More recently, hybrid architectures have emerged. Zhang et al. proposed the hybrid deep learning architecture YOLO-SWFormer, integrating Swin Transformer backbones into the YOLO framework to capture global dependencies and suppress sea clutter. Their method achieved a recall rate of 91.8% on the evaluated PPI radar dataset [9]. For resource-limited deployment scenarios, Kang et al. introduced YOSMR, a lightweight detection network built upon a MobileNetV3 backbone and depthwise separable convolutions. The proposed framework was specifically optimized for nearshore ship detection and achieved a recall of 93.08%, an accuracy of 92.04%, and a precision of 92.15% [10].

Handling sea clutter remains a challenging sub-field within deep learning research. Generative Adversarial Networks (GANs) have been applied for this purpose. Mou et al. proposed SCS-GAN, utilizing residual attention mechanisms to suppress clutter while preserving target integrity [11]. Building on this, Pei et al. introduced a cyclic GAN architecture with a target-consistency loss to perform image-to-image translation from cluttered to clean radar data. The proposed architecture learns bidirectional mappings between cluttered and clutter-free domains, enabling robust feature learning [12].

Beyond single-sensor detection, multi-sensor fusion strategies have been explored to enhance robustness. Xu et al. presented a multistage fusion framework, which combines marine radar data and camera output, utilizing a Transformer-based module to bridge the semantic gap between modalities. Their approach reported a detection performance of 88.56% mAP@0.50 [13]. Similarly, Liu et al. developed a coarse-to-fine hybrid recognition system to achieve robust ship identification for inland waterways, fusing radar Regions of Interest (ROIs) with CCTV images using a target-free calibration method. Authors developed a custom lightweight CNN called VesNet that performs feature extraction and classification [14].

Accurate detection forms the foundation of effective and reliable tracking, where several distinct approaches have been proposed. Vo et al. improved plot-level processing using Modified Fuzzy C-Means clustering to estimate target centroids before Kalman filtering [15]. Additionally, for X-band marine radar, a combined particle-Kalman filter approach was proposed and successfully tested for maneuvering target tracking from nautical radar images [16]. Hu et al. proposed a multi-task network that simultaneously performs detection and appearance feature embedding, enhanced by cascading consecutive radar scans [17]. Kim et al. integrated deep learning uncertainty into tracking by using Gaussian YOLOv3 outputs within an Extended Kalman Filter (EKF) framework [18]. For autonomous surface vehicles, Kim et al. developed DPSE-Net for four-class semantic segmentation coupled with a radar-specific DeepSORT tracker [19]. Addressing the geometry of vessels, Fowdur’s doctoral work focused on multiple extended target tracking (METT), modeling ships as ellipses and using Joint Probabilistic Data Association (JPDA) [20]. Most recently, Chen et al. introduced ShipMOT, a framework featuring a Nonlinear State Augmentation filter and a dual-criteria association metric to handle dense traffic and non-uniform motion. Authors use Bounding Box Similarity Index (BBSI) for data association that combines geometric shape correlation and centroid alignment [21].

A persistent challenge in marine radar object detection is the resolution mismatch between PPI images and standard network input sizes. This leads to the detection loss of small targets. To address this in general computer vision, Akyon et al. introduced Slicing Aided Hyper Inference (SAHI), a technique that slices high-resolution images during inference to improve small object detection without retraining the model. SAHI operates by systematically slicing large input images into overlapping smaller patches, performing object detection on each slice independently, and then merging the detections back to the full image [22].

The primary objective of this study is to achieve high-precision, orientation-aware detection and precise silhouette delineation of marine vessels in real-world PPI radar imagery under challenging conditions, including small/distant targets, sea clutter, wake trails, and interference from navigational aids. While simple color-based thresholding can separate prominent echoes in low-clutter frames it fails in realistic scenarios with variable signal-to-noise ratios and overlapping returns. Therefore, this work goes beyond binary target detection to provide accurate centroid localization (sub-10-pixel error), using orientation-aware bounding (via OBB).

In this study, we propose a high-precision detection pipeline that combines tiled training, SAHI, and the YOLOv11-OBB architecture. While previous works rely on axis-aligned bounding boxes or standard resizing methods, our approach explicitly addresses scale variance and target orientation. By integrating oriented bounding boxes with slicing strategies, we aim to achieve better localization and detection performance. This work presents the first unified investigation of tiled training combined with SAHI and oriented bounding box detection for shipborne marine radar data, with quantitative validation against AIS-derived ground truth. The main contributions of this study to literature can be summarized as follows:

- We propose a specialized detection pipeline that integrates tiled training and SAHI with the YOLOv11-OBB architecture. This approach effectively resolves the resolution mismatch in large-scale PPI images, significantly improving the detection of small targets compared to traditional full-image resizing methods.
- The experiments indicate that OBBs provide better localization accuracy and clutter rejection for marine radar targets. By explicitly modeling the orientation of extended vessel echoes, our method reduces false positives caused by sea clutter and fixed aids to navigation, yielding tighter and more reliable target bounds.
- We introduce a robust semi-automatic contour-based annotation strategy that generates consistent multi-format labels (axis-aligned, oriented, and segmentation) directly from raw radar returns.
- The proposed framework is validated on the real-world dataset, achieving over 0.95 mAP@0.5 and sub-10-pixel localization error. These results establish a new benchmark for high-precision marine radar object detection that remains computationally efficient enough for near-real-time deployment on edge hardware.

The remainder of this paper is organized as follows. Section 2 details the materials and methods, including the characteristics of the dataset, the proposed semi-automatic annotation pipeline, and the implementation specifics of the tiled training and SAHI strategies. Section 3 presents experimental results, providing a comparison of the YOLOv11 variants, an assessment of detection performance, and an in-depth error analysis. Section 4 discusses the implications of these findings, evaluates the computational efficiency of the pipeline, and outlines potential directions for future research.

## 2. Materials and Methods

### 2.1. Dataset

The experiments in this study use the DAAN dataset from the real-world marine radar repository provided by the German Aerospace Centre (DLR). This repository contains three datasets (DAAN, DARC, and MANV) collected during measurement campaigns in the Baltic Sea, specifically designed for evaluating target detection and multiple target tracking algorithms in maritime environments [23]. The DAAN dataset was selected due to its characteristics, including the presence of multiple vessels (4 targets), dynamic own-ship motion, radar trails from vessel wakes, and interference from fixed aids to navigation. These elements introduce realistic complexities, making it particularly suitable for assessing high-precision object detection methods on marine radar images.

The sensor is a shipborne X-band marine radar (non-coherent, non-Doppler pulse waveform) mounted on the own-ship platform. It operates in standard nautical PPI mode with a nominal 3 NM range scale and 1 Hz frame capture frequency via screen grabbing. No Doppler processing or moving target indication (MTI) is applied in the acquired imagery. The radar is a commercial maritime navigation radar (specific model not disclosed in the DLR repository), typical of those installed on small to medium-sized vessels.

The radar used is mounted on the dynamic own-ship (Vessel 1), with screen visuals captured at approximately 1 Hz using frame grabbers. Images were processed by cropping user-interface elements to focus on the region of interest, and raw radar data are provided in the form of polar images as described in the original repository. Ground-truth reference trajectories for the vessels are derived from synchronous Automatic Identification System (AIS) data, providing accurate position labels (in meters, North-East coordinates) for evaluation purposes.

**Figure 1:**
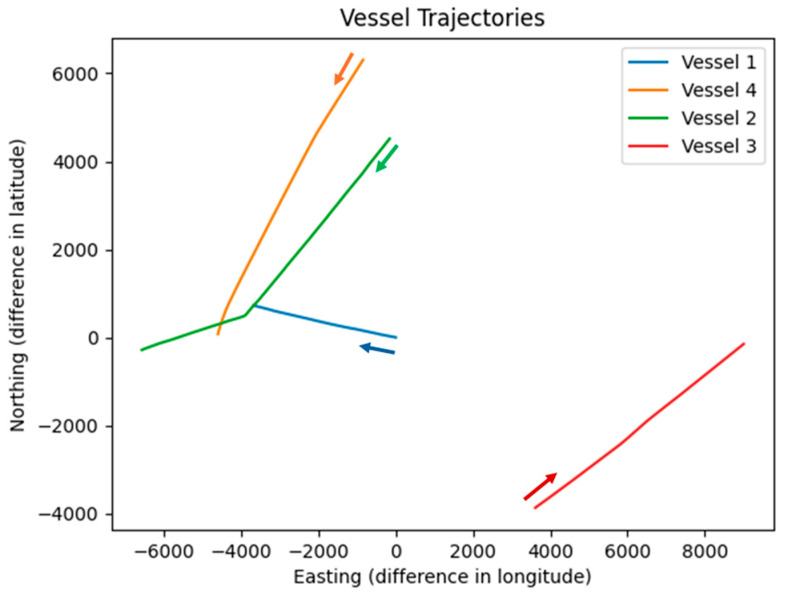
Absolute vessel trajectories in the North-East coordinate system during the dataset collection period. Colored lines represent the paths of the four target vessels derived from synchronous AIS data. Arrows indicate the direction of motion at selected time steps.

The absolute positions of the four vessels are visualized in Figure 1. Trajectories are plotted in a North-East reference frame using ground-truth AIS data recorded synchronously with the radar measurements. Each vessel is represented by a distinct color, with directional arrows overlaid at representative points along the paths. The plot clearly illustrates the relative motion of the targets with respect to the own-ship platform throughout the 13-min observation period.

The trajectories shown in Figure 1 highlight the complexity of the real-world maritime scenario. Vessels exhibit significantly different speeds, headings, and encounter geometries, including crossing situations and overtaking maneuvers. These diverse motion patterns and target orientations underscore the necessity of orientation-aware methods, such as YOLOv11-OBB, to achieve robust detection in marine radar environments.

**Figure 2:**
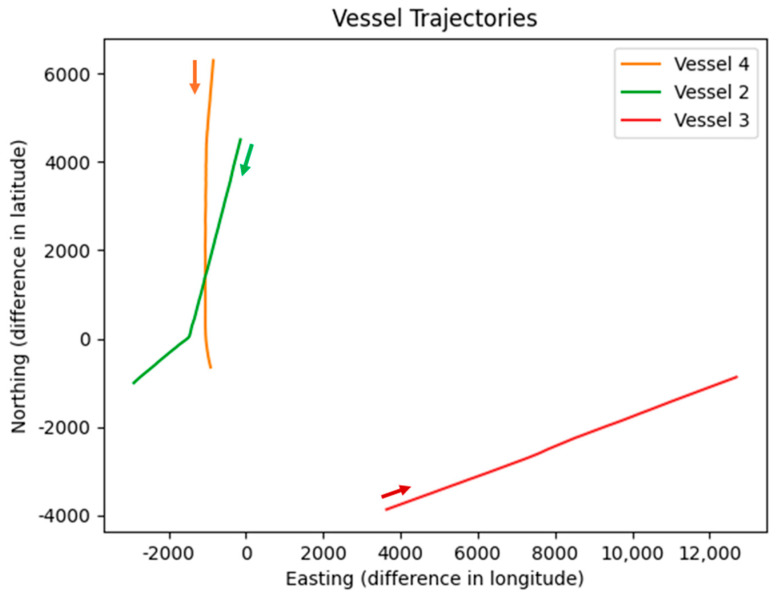
Relative vessel trajectories with respect to Vessel 1 (own-ship) in the North-East coordinate system. Colored lines show the motion paths of the three target vessels derived from AIS data. Arrows indicate the direction of motion at representative time steps.

Figure 2 presents the vessel trajectories relative to Vessel 1, which serves as the moving own-ship platform. Positions are expressed in a North-East coordinate frame centered on Vessel 1. Each target vessel is depicted by a unique color, with directional arrows marking the sense of motion.

The relative trajectories in Figure 2 provide a radar-centric view, mirroring the perspective of the PPI images where targets are positioned relative to the own-ship at the center. This visualization reveals key dynamics, such as varying relative bearings, ranges, and closure rates, which directly influence radar echo shapes and orientations. For instance, approaching vessels may exhibit elongated echoes due to increasing signal strength, while crossing targets show pronounced orientation changes. These insights justify the emphasis on scale-aware (tiled training and SAHI) and orientation-aware (OBB) strategies in our pipeline, as they address the dataset’s inherent variabilities in target appearance from the sensor’s viewpoint.

### 2.2. Semi-Automatic Data Annotation

The DAAN dataset includes synchronous AIS data providing accurate positional ground truth (in North-East coordinates) for vessel trajectories, which is primarily used for evaluation purposes, such as centroid localization accuracy. AIS alone does not capture the visual characteristics of radar echoes in the PPI images. Radar returns exhibit variable shapes, extents, and orientations due to factors like vessel aspect angle, wake trails, and clutter, necessitating pixel-level labels (bounding boxes, OBBs, and masks) derived directly from image intensities.

To enable supervised training of the multi-task YOLOv11 architectures (YOLOv11-detect, YOLOv11-seg, and YOLOv11-obb), we developed a semi-automatic annotation pipeline. This pipeline converts raw radar echoes into precise, multi-format labels, addressing the specific challenges of marine radar imagery. The process consists of three stages: (1) Signal-based candidate extraction, (2) Geometric label generation, and (3) Human-in-the-loop verification.

**Figure 3:**
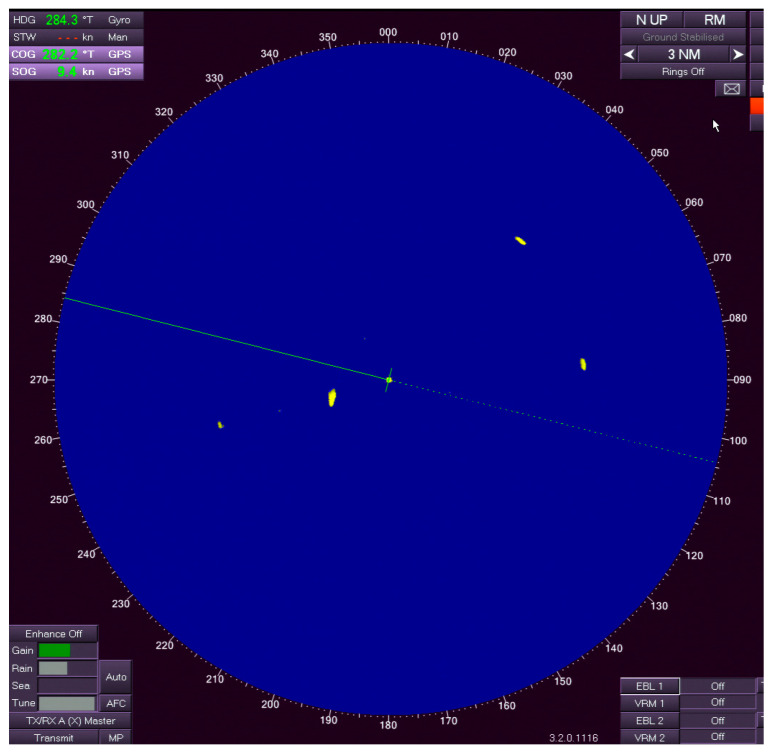
Example of a full-resolution PPI radar image (760.png) from the dataset [23]. The image shows the raw marine radar display in relative motion mode (North Up, Range 3 NM), including multiple vessel echoes, extended wake trails, sea clutter, and fixed aids to navigation.

Figure 3 presents a representative PPI radar image from the dataset. The display is in North-Up, relative motion mode with a 3 NM range scale. Bright yellow echoes correspond to vessel targets. Several small and medium-sized targets are visible at different bearings and ranges. The image also contains low-level sea clutter and occasional interference from aids to navigation. Small distant vessels appear as low-intensity, compact returns. Sea clutter and fixed aids add persistent background noise.

#### 2.2.1. Automated Candidate Extraction

Given the distinct color representation of the PPI images, where high-intensity returns (targets) are rendered in yellow against a dark background, we employed a color-space segmentation approach to isolate potential vessel targets. Let *I* be the input RGB radar image with dimensions *H × W*. The original RGB image is converted to the Hue-Saturation-Value (HSV) color space so that thresholding can specifically target the bright yellow color used by marine radar PPI displays to highlight strong vessel echoes. A binary mask M_bin_ is generated by thresholding the HSV channels specifically for the yellow spectrum characteristic of the radar echoes, as given in Equation (1).

M_bin_ (x,y) = 1 if h ∈ [20, 40] ∧ s ∈ [100, 255] ∧ v ∈ [100, 255], else 0 (1)

Morphological filtering is applied to extract connected components (contours). Let C = {c_1_, c_2_, …, c_n_} be the set of detected contours. To mitigate noise from sea clutter, a spatial filter removes any contour c_i_ where the area A (c_i_) < τ_area, with τ_area = 8 pixels.

#### 2.2.2. Multi-Format Label Generation

For every valid contour c_i_, three distinct label formats are computed simultaneously to satisfy the input requirements of the YOLOv11 variants:

1. Axis-Aligned Bounding Box (AABB): For the standard detection task, we compute the minimal up-right bounding rectangle. This is defined by the tuple (x_c_, y_c_, w, h), where (x_c_, y_c_) is the normalized center, and w and h are the normalized width and height.
2. Instance Segmentation Mask: To capture the irregular shapes of targets, the contour c_i_ is approximated to a polygon P_i_ using the Douglas-Peucker algorithm. The approximation accuracy ε is dynamically set to 1% of the contour perimeter S_arc_(c_i_). The perimeter S_arc_(c_i_) is computed as the arc length of the contour, calculated by summing the Euclidean distances between consecutive points in the contour point set.

ε = 0.01 · S_arc_(c_i_) (2)

This reduces vertex redundancy while preserving the geometric topology of the radar echo.

3. Oriented Bounding Box (OBB): To address the orientation sensitivity of marine targets, we compute the minimum area rotated rectangle enclosing the point set of c_i_. This is solved as an optimization problem to find the rectangle R_θ_ with rotation θ that minimizes area. The OBB is represented by four normalized corner points O_pts_ = [(x_1_, y_1_), (x_2_, y_2_), (x_3_, y_3_), (x_4_, y_4_)], ensuring that the label encompasses the target’s physical orientation rather than its axis-aligned projection.

The automated generation process is formalized in Algorithm 1.
**Algorithm 1.** Semi-automatic multi-label generation for marine radar images.**Input:** Radar Image I, Threshold $T_{hsv}$, Area Threshold τ**Output:** Set of annotations $L_{det},L_{seg},L_{obb}$**1:** $I_{hsv}\leftarrow$ RGB2HSV (I)**2:** M ← InRange $(I_{hsv},T_{lower},T_{upper}$)**3:** C ← FindContours (M)**4: for each** contour c∈C **do****5:**  area ← ContourArea (c)**6:**  if area <τ **then****7:    continue****8:  end if****9:**  $\left(x,y,w,h\right)\leftarrow$ BoundingRect (c)**10:**    $L_{det}\leftarrow$ Normalize (x,y,w,h)**11:**    ϵ ←0.01 × ArcLength (c)**12:**    poly ← ApproxPolyDP (c,ϵ)**13:**    $L_{seg}\leftarrow$ Normalize (poly)**14:**    $rect_{rot}\leftarrow$ MinAreaRect (c)**15:**    $box_{pts}\leftarrow$ BoxPoints $(rect_{rot}$)**16:**    $L_{obb}\leftarrow$ Normalize $(box_{pts}$)**17:** 
**end for**
**18:** 
**return** 
$L_{det},L_{seg},L_{obb}$


The chosen HSV thresholds for the yellow spectrum were empirically determined by analyzing a representative subset of the DAAN dataset, where high-intensity vessel echoes consistently appear in this narrow yellow band due to the radar display’s standardized color mapping for strong returns (bright yellow against dark background). This mapping is largely invariant to minor gain or lighting adjustments on the radar screen, as the PPI images are captured post-display processing with fixed color lookup tables typical in commercial marine radars. Fixed global thresholds proved sufficient for this dataset because: (i) the radar display uses consistent color encoding for echo intensity, (ii) frames exhibit relatively uniform background (dark sea), and (iii) only ~15% of frames required manual correction for threshold failures. Nevertheless, we acknowledge that significant gain changes or varying screen brightness could shift the effective HSV ranges. In such cases, adaptive thresholding or dynamic HSV range estimation per frame could further improve robustness.

#### 2.2.3. Consistency Refinement and Manual Verification

Finally, a human-in-the-loop verification step was introduced to ensure annotation quality. Approximately 15% of the frames were manually edited to address semantic errors that automatic thresholding alone could not resolve. This manual quality-control step was performed only once, during the creation of the ground-truth labels. Importantly, this human intervention is confined to the offline dataset annotation phase and does not affect the runtime performance of the trained model. Once the model is trained on the verified labels, inference on new radar frames is fully automatic, requiring no manual input. Corrections primarily involved splitting merged contours of distinct vessels, merging fragmented echoes from the same vessel, and removing false positives arising from static aids to navigation with intensity profiles like vessels. This combination of automated contour detection and targeted human refinement produced a high-fidelity dataset with consistent and accurate annotations across all three formats: axis-aligned bounding boxes, instance segmentation masks, and oriented bounding boxes (OBBs). Two training datasets were then prepared:

- Full-image dataset: Original PPI images were resized to 640 × 640 pixels, and all labels (bbox, obb, seg) were correspondingly scaled.
- Tiled dataset: To improve detection of small and distant targets, full-resolution images were divided into 640 × 640 overlapping tiles (20% overlap) using a custom script. Only tiles containing at least one object (based on polygon centroid) were retained, and labels were cropped and re-normalized relative to each tile. This significantly augmented the number of positive samples containing detectable targets.

The final dataset was split temporally into training (80%) and test (20%) sets to avoid overlapping between consecutive frames of the same vessel trajectories. A single class (“target”) was defined, encompassing all vessel types and sizes.

### 2.3. YOLOv11 Models

This study employs three variants of the Ultralytics YOLOv11 architecture, which represents the latest advancement in the YOLO series for real-time object detection and related tasks. YOLOv11 builds upon previous versions by incorporating an optimized backbone and neck design, primarily utilizing C3k2 blocks (an efficient evolution of the C2f modules) for enhanced feature extraction, along with components such as Spatial Pyramid Pooling Fast (SPPF) and attention mechanisms (C2PSA) to improve multi-scale feature fusion. The detection head supports multiple tasks through a decoupled, anchor-free design, enabling high accuracy with reduced parameters and computational overhead compared to earlier models [24]. The selected variants are:

- YOLOv11-detect: Performs standard object detection using axis-aligned bounding boxes (AABB). It predicts class probabilities and bounding box coordinates (center_x, center_y, width, height) for each detected object.
- YOLOv11-seg: Extends detection to instance segmentation, simultaneously outputting bounding boxes and pixel-level masks for precise delineation of object boundaries.
- YOLOv11-obb: Supports oriented object detection (OBB), predicting rotated bounding boxes defined by four corner points.

These models were chosen for their suitability to the challenges of marine radar imagery, including extended and elongated target echoes, variable vessel orientations due to heading changes, low signal-to-noise ratios from clutter, and the presence of small targets. The OBB variant addresses orientation variability without relying on axis-aligned approximations, while the segmentation variant captures irregular shapes of radar returns more accurately than bounding boxes alone. In this context, oriented bounding boxes and instance segmentation are particularly valuable, as they provide a tighter geometric fit and explicit silhouette information that cannot be captured by conventional axis-aligned bounding boxes. The enhanced feature extraction in YOLOv11’s backbone and neck improves robustness to dense clutter, enabling better discrimination of true vessels in complex PPI scenes. All models benefit from transfer learning via COCO pretrained weights, facilitating effective fine-tuning on the dataset.

### 2.4. Training Procedure

#### 2.4.1. Full-Image Training

All models were trained using the Ultralytics YOLOv11 implementation (version 8.3.235) on a system equipped with an NVIDIA GeForce RTX 3050 6 GB Laptop GPU, Python 3.13.2, and PyTorch 2.7.1 with CUDA support. Training was conducted separately for the three tasks: detection (YOLOv11n-detect), oriented bounding box detection (YOLOv11n-obb), and instance segmentation (YOLOv11n-seg).

Common hyperparameters were kept consistent across all models to ensure fair comparison and reproducibility. The batch size was set to 16, and training was conducted for 50 epochs. The AdamW optimizer was used in automatic mode. The initial learning rate was 0.01, with cosine annealing applied to reach a final value of 0.01 × lrf. Image augmentations included mosaic = 1.0, fliplr = 0.5, hsv_h = 0.015, hsv_s = 0.7, hsv_v = 0.4, translate = 0.1, scale = 0.5, and erasing = 0.4. Loss weights were configured as box = 7.5, cls = 0.5, and dfl = 1.5. Non-maximum suppression employed an IoU threshold of 0.7.

#### 2.4.2. Tiled Training

To address the limitations of full-image training, particularly the loss of resolution for small targets when resizing images to 640 × 640 pixels, a tiled training approach was implemented. This strategy significantly augments the dataset by creating multiple high-resolution 640 × 640-pixel tiles from each original full-resolution image, thereby improving the representation of small objects and increasing the number of positive training samples.

Tiling was performed using a custom script that divides each annotated PPI image into overlapping tiles with a 20% overlap ratio (step size = 640 × 0.8 = 512 pixels). To ensure full coverage at image borders, tiles smaller than 640 × 640 were padded by shifting the crop window accordingly. Only tiles containing at least one object were retained. Labels were then cropped to the tile coordinates and re-normalized to the 640 × 640 tile space, preserving compatibility with YOLOv11-detect, YOLOv11-obb, and YOLOv11-seg formats.

This process substantially expanded the dataset size: the tiled dataset comprised 3040 training images with 11,893 labeled instances, compared to the original full-image dataset (760 images, 4457 instances). The increased sample diversity, especially for smaller targets, contributed to improved model robustness against scale variations typical in marine radar scenes. Training hyperparameters and settings were identical to those used in full-image training, including the use of COCO-pretrained weights, 50 epochs, batch size of 16, and the same augmentation pipeline. The same hardware configuration and Ultralytics version 8.3.235 were employed.

### 2.5. SAHI Strategy

To further enhance detection performance on full-resolution marine radar PPI images, a test-time augmentation strategy known as SAHI [21] was employed. SAHI addresses the resolution mismatch between training (640 × 640 pixels) and inference on large, high-range radar images by performing sliced inference followed by intelligent result merging.

SAHI was implemented using the official SAHI library, applied to the best-trained models obtained from both full-image and tiled training approaches. For each YOLOv11n variant (detect, OBB, and segmentation), inference was performed on the original full-resolution test images. A slice size of 640 × 640 pixels was used to match the training resolution, with an overlap ratio of 20% to ensure smooth merging and minimize boundary artifacts. Post-processing employed class-agnostic non-maximum suppression (NMS) with a match threshold of 0.5 to effectively combine overlapping predictions across slices. A confidence threshold of 0.5 was applied consistently across all variants.

### 2.6. Evaluation Metrics

Performance evaluation was conducted using the standard COCO object detection metrics implemented via the pycocotools library. All metrics were computed on the held-out test set consisting of full-resolution PPI images, with predictions generated under three inference strategies: direct full-image inference, direct tiled-model inference on full images (resized to 640 × 640), and tiled-model inference with SAHI. The primary metrics reported are:

- mAP@0.5: Mean Average Precision at IoU threshold 0.5 (primary ranking metric for marine radar detection, emphasizing correct localization within reasonable tolerance).
- mAP@0.5:0.95: Mean Average Precision averaged over IoU thresholds from 0.5 to 0.95 (in steps of 0.05), providing a more comprehensive measure of localization accuracy across varying strictness levels.
- Precision (P) and Recall (R): Reported at IoU = 0.5 to assess false positive and false negative rates.

## 3. Results

### 3.1. Comparison of Detection Performance Across Lightweight YOLO Variants

To evaluate the suitability of various lightweight YOLO architectures for marine radar object detection, we conducted a comparative analysis across multiple YOLO variants, focusing on their performance metrics, computational efficiency, and model complexity. This comparison is essential for identifying the optimal backbone that balances high accuracy with low resource demands. The selected variants include YOLOv5n [25], YOLOv8n [26], YOLO11n [24], and YOLO26n [27], all trained under identical conditions on the DAAN dataset using tiled training. Results are given in Table 1.

The results in Table 1 show that all variants achieve high mAP@0.5 scores above 0.98, indicating strong overall detection capability. YOLO11n stands out with the highest mAP@0.5:0.95 of 0.816, achieving better detection accuracy. This metric is important for marine radar applications, where precise bounding of small and oriented targets is required. Although YOLO26n offers the lowest GFLOPs and parameter count for potential efficiency gains, YOLO11n provides a favorable trade-off with higher inference time (2.9 ms) and computational load, justifying its selection for subsequent experiments involving tiled training, SAHI, and OBB enhancements.

### 3.2. Comparison of Full-Image Resizing and Tiled Training

Table 2 presents the performance metrics for three YOLOv11 architectures. It compares standard full-image resizing against the proposed tiled training strategy. The evaluation uses mAP@0.5 and mAP@0.5:0.95 metrics. The tiled approach yields consistently higher scores for all model variants. Specifically, the YOLOv11-obb model with tiled training achieves the highest accuracy. It reaches an mAP@0.5 of 0.989. This shows the quantitative impact of the training resolution on model performance.

These results highlight the critical limitations of standard image resizing. Resizing leads to significant information loss for small radar targets. Tiled training effectively addresses this resolution mismatch. Consequently, detection metrics improve significantly across all tasks. The OBB variant outperforms the standard detection and segmentation models. This confirms the importance of orientation-aware learning for marine vessels. Furthermore, the superior mAP@0.5:0.95 score indicates tighter and more precise target localization.

### 3.3. SAHI-Based Comparative Results

To investigate the impact of the overlap ratio in SAHI on detection performance, additional experiments were conducted using the YOLOv11-detect model (tiled training variant). The slice size was fixed at 640 × 640 pixels to match the training resolution, while the overlap ratio varied from 0.1 to 0.5. All other SAHI parameters, including NMS match threshold (0.5) and confidence threshold (0.5), remain consistent. The mean Average Precision (mAP) was computed at IoU thresholds of 0.5, 0.5:0.95, and 0.75. Results are given in Table 3.

Increasing the overlap ratio from 0.1 to 0.5 yields a modest but consistent improvement in localization precision, particularly at stricter IoU thresholds. The mAP@0.5 remains stable across configurations, indicating robust target identification. However, mAP@0.5:0.95 rises gradually from 0.4867 to 0.4896, and mAP@0.75 increases more noticeably from 0.3803 to 0.3941. These gains are attributed to reduced boundary artifacts and better merging of overlapping predictions via NMS, which enhances precise bounding for small and elongated radar echoes. Higher overlap ratios improve performance on challenging cases without significantly degrading mAP@0.5 or increasing false positives. The original 0.2 overlap provides a good trade-off between accuracy and computational cost, while ratios of 0.4–0.5 offer further refinement for applications prioritizing localization accuracy.

To investigate the impact of the slice size in SAHI on detection performance, additional experiments were conducted using the YOLOv11-detect model (tiled training variant). The overlap ratio was fixed at 0.2, while the slice size varied from 640 px down to 240 px. All other SAHI parameters, including NMS match threshold (0.5) and confidence threshold (0.5), remain consistent. The mean Average Precision (mAP) was computed at IoU thresholds of 0.5, 0.5:0.95, and 0.75. Results are given in Table 4.

The results in Table 4 show interesting trends in how slice size affects detection performance. As slice size decreases from 640 px to 320 px, there is a general improvement in mAP metrics, particularly at stricter IoU thresholds like mAP@0.75, which rises from 0.3811 to 0.4860, indicating enhanced localization precision for small and elongated radar echoes due to finer-grained slicing that better captures scale variations. However, further reduction to 240 px shows a decline in mAP@0.5:0.95 and mAP@0.75, suggesting a point of diminishing returns, where excessive slicing introduces more boundary artifacts or redundancy in merging predictions. Notably, as slice size decreases, the number of slices processed increases more than linearly. For instance, halving the slice size roughly quadruples the slice count due to the two-dimensional nature of image tiling. Smaller slice size leads to higher computational overhead that must be balanced against accuracy gains for real-time applications.

Table 5 presents the detection performance of the three YOLOv11 variants. We evaluate the models using three distinct metrics: mAP@0.5, mAP@0.5:0.95, and mAP@0.75. The YOLOv11-detect model achieves the highest mAP@0.5 score of 0.9623. However, its performance drops significantly at stricter thresholds. The YOLOv11-seg model achieves the highest precision, indicating more accurate localization. It obtains an mAP@0.5:0.95 of 0.4937 and an mAP@0.75 of 0.4411. The YOLOv11-obb model follows closely with an mAP@0.75 of 0.4281.

These results indicate that all models are highly effective at standard detection levels. The high mAP@0.5 scores confirm robust target identification. However, the performance at higher thresholds reveals important differences. The standard detection model struggles with precise localization. Its score drops to 0.3811 at mAP@0.75. In contrast, the OBB and Segmentation models maintain higher accuracy at this strict threshold. This validates the advantage of orientation-aware and pixel-level methods. They provide tighter and more accurate bounding boxes for marine vessels.

To evaluate the trade-off between detection completeness and accuracy across YOLOv11 variants, we computed precision and recall metrics at an IoU threshold of 0.5, as given in Table 6. These metrics are derived from true positives (TP), false positives (FP), and false negatives (FN). Precision measures the proportion of correct detections among all predicted positives, while recall assesses the fraction of ground-truth targets successfully detected. This analysis complements mAP by highlighting model-specific error tendencies in marine radar scenes.

The analysis of Table 6 presents nuanced performance differences among YOLOv11 variants. YOLOv11-detect achieves the highest precision (0.9290) and recall (0.9576), driven by fewer false positives (326) and false negatives (189), suggesting strong overall accuracy in target identification and clutter suppression. YOLOv11-obb shows slightly lower precision (0.9123) and recall (0.9545), with increased false positives (409) and false negatives (203), indicating a modest trade-off, possibly due to its focus on orientation modeling, which may introduce errors in bounding elongated echoes. YOLOv11-seg exhibits the lowest precision (0.8728) from high false positives (620), implying greater susceptibility to over-detection, though its recall (0.9549) remains competitive.

Although the YOLOv11-obb variant generates more false positives (409) compared to YOLOv11-detect (326), evaluations incorporate real AIS data for validation, ensuring only AIS-compatible detections are prioritized. This filtering mitigates the impact of extraneous positives from clutter or wakes. Consequently, despite the YOLOv11-obb model’s modestly lower precision (0.9123) and recall (0.9545), it excels in centroid localization by better aligning with true vessel orientations, as corroborated by subsequent error analyses showing reduced mean distances to AIS ground truths.

### 3.4. Centroid Localization Accuracy Analysis

Table 7 presents a detailed analysis of localization accuracy by quantifying the Euclidean distance errors between the centers of predicted bounding boxes and their corresponding ground-truth AIS-based points for correctly detected vessels in the dataset. Evaluations were performed on detections obtained from the best-performing configuration (tiled training followed by SAHI) with a confidence threshold of 0.5 and IoU ≥ 0.5 for matching. Only Vessel 2 and Vessel 4 were considered in this analysis, as they provided enough detections across all frames and model variants to support a meaningful statistical comparison. Vessel 1 corresponds to the own-ship. Vessel 3 exhibited intermittent visibility resulting from its range and trajectory characteristics. The metrics include mean, median, minimum, maximum, and standard deviation of the center errors in pixels on full-resolution PPI images. Lower values indicate higher localization precision.

The results in Table 7 indicate that all three YOLOv11 variants achieve sub-10-pixel mean center localization error for both analyzed vessels, with the OBB model consistently showing the lowest mean and median errors, confirming its advantage in precisely capturing the orientation and extent of elongated radar echoes even under challenging conditions.

### 3.5. Computational Efficiency

All experiments were conducted under identical hardware conditions to ensure fair and reproducible comparisons. The lightweight YOLOv11n backbone was used consistently across all variants, with model sizes of approximately 5.5 MB for YOLOv11n-detect, 5.9 MB for YOLOv11n-obb, and 6.0 MB for YOLOv11n-seg. Peak memory consumption remained below 4.2 GB in all configurations, which is well within the resource limits of typical edge hardware deployed on small to medium-sized vessels.

Full-image inference on 1050 × 1024-pixel radar frames achieved 18–22 frames per second across the detection, segmentation, and oriented bounding box models. When SAHI was applied to full-resolution PPI images, the tiled processing approach reduced inference speed. The proposed tiled YOLOv11n-OBB model with SAHI reached 4–6 FPS, representing a 3–4× slowdown compared to the full-image baseline. Despite this reduction in frame rate, the performance remains highly practical for maritime applications. Operation at 4–6 FPS adequately supports near-real-time tasks such as collision avoidance, small-vessel situational awareness, and integration with AIS-based tracking systems. These safety-critical applications prioritize high detection accuracy and precise localization over maximum throughput.

In summary, the proposed pipeline achieves a favorable trade-off. It delivers substantial improvements in accuracy (>0.95 mAP@0.5) and localization precision (sub-10-pixel mean error) while maintaining sufficient computational efficiency for real-world onboard deployment in marine radar systems.

### 3.6. Visual Comparison of Ground Truth and Model Predictions

**Figure 4:**
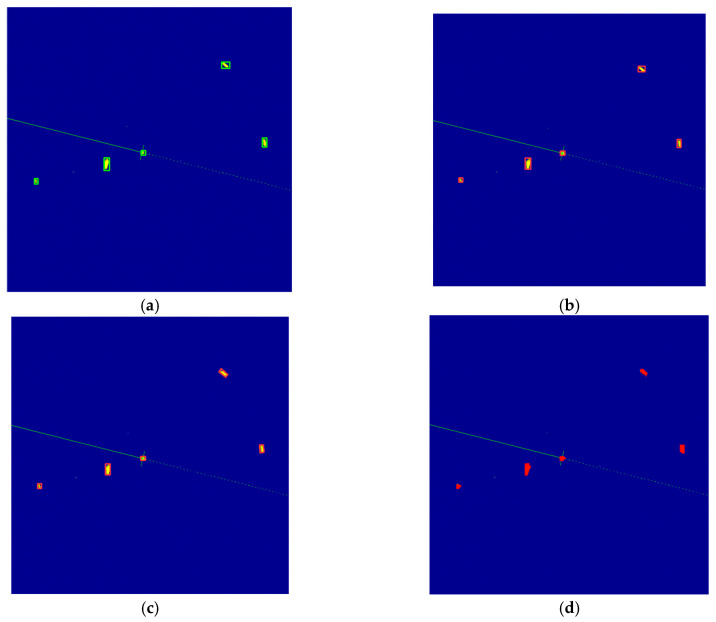
Qualitative comparison of ground truth and model predictions on a sample frame from the dataset. (**a**) Ground-truth annotations derived from semi-automatic contour-based labeling of radar echoes, showing axis-aligned bounding boxes. Predictions from the tiled YOLOv11n-detect model with SAHI, (**b**) using axis-aligned bounding boxes, (**c**) using oriented bounding boxes, (**d**) using instance segmentation masks.

Figure 4 illustrates the ground-truth annotations and corresponding predictions from the three YOLOv11 variants on a selected frame that contains multiple small and distant targets. This image represents one of the cases where the OBB model outperforms the other variants in accurately detecting the position of the targets. The frame was processed using the proposed tiled training and SAHI pipeline (640 × 640 slice size, 20% overlap) on full-resolution PPI images. Ground-truth labels (Figure 4a) were generated from color-based contour detection and manual corrections, encompassing all significant radar returns classified as “target”. Figure 4b–d show post-NMS predictions at a confidence threshold of 0.5, overlaid on the original radar PPI image for direct visual comparison.

The visual comparison in Figure 4 highlights the improvements achieved by incorporating orientation and segmentation capabilities in YOLOv11 architecture. While the standard detection (Figure 4b) successfully localizes most targets with axis-aligned bounding boxes, it exhibits noticeable misalignment on elongated echoes, leading to larger bounding boxes. In contrast, the OBB model (Figure 4c) provides tighter and more accurate fits by adapting to the arbitrary orientations of the vessel, reducing overlap with surrounding clutter. The segmentation variant (Figure 4d) further refines this by delineating precise instance masks, effectively separating overlapping wakes from primary vessel echoes and suppressing spurious returns near aids to navigation. Notably, all models correctly detect the small, low-intensity targets that are challenging due to their size and proximity to noise, demonstrating the efficacy of tiled training and SAHI in overcoming scale limitations inherent to full-image approaches.

**Figure 5:**
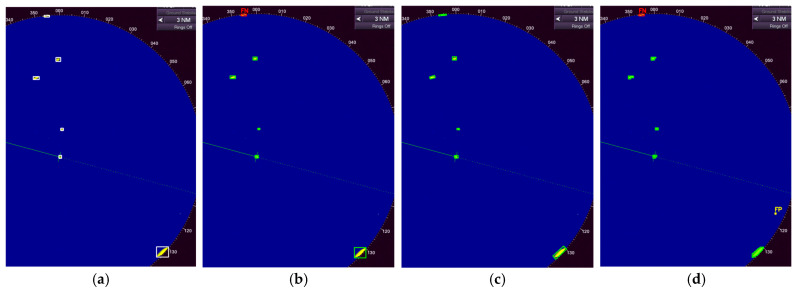
Example comparison of ground truth and model predictions on a sample frame (069.png) from the dataset. (**a**) Ground-truth annotations shown in white, derived from semi-automatic contour-based labeling of radar echoes. Predictions from the tiled YOLOv11n models with SAHI: (**b**) using axis-aligned bounding boxes, (**c**) using oriented bounding boxes, (**d**) using instance segmentation masks. True positives are marked in green, false positives in yellow, and false negatives in red.

Figure 5 presents a comparison between the ground-truth annotations and the predictions generated by three variants of the YOLOv11n model. The ground-truth labels (depicted in white) were derived from a semi-automatic contour-based annotation process that identifies significant radar echoes classified as targets. Model predictions are visualized using color-coded markers: green for true positives (correct detections aligning with ground truth), yellow for false positives (erroneous detections of non-targets), and red for false negatives (missed targets).

### 3.7. Analysis of Typical Failure Cases

**Figure 6:**
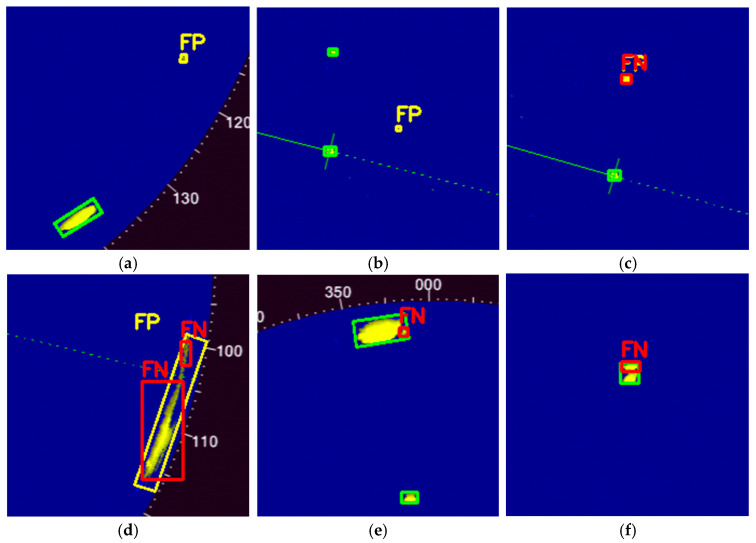
Representative examples of typical failure cases in the proposed pipeline, highlighting false negatives (FN) and false positives (FP). (**a**) FN due to environmental effects, such as signal attenuation or scattering. (**b**) FN caused by wave clutter, where sea states obscure the vessel’s signature. (**c**) Combined FP and FN from SAHI slicing, with patch division disrupting feature aggregation, leading to IoU mismatches. (**d**) FN induced by target motion, where maneuvers alter echo shapes through aspect angle shifts, evading model detection. (**e**) FP where a single target is split into two due to Doppler effects or reflections during maneuvers. (**f**) FN from overlapping targets, where echoes of two vessels merge, causing the OBB head to undercount in dense traffic scenarios.

Despite the high mean average precision (mAP@0.5 > 0.95) and low localization errors achieved by the proposed model, certain challenging scenarios in marine radar imagery can lead to detection failures. These failures primarily manifest as false positives (FPs), where non-target echoes such as sea clutter or interference are incorrectly identified as vessels, and false negatives (FNs), where actual vessels are missed due to low signal-to-noise ratio (SNR), occlusion, or extreme scale variations. Analyzing these cases is essential for understanding the limitations of the current pipeline and guiding future enhancements, such as improved clutter suppression or multi-modal fusion. In this section, we examine six representative failure cases drawn from the dataset, categorized by error type and illustrated in Figure 6. Each case highlights specific environmental or algorithmic factors that cause errors.

Figure 6a illustrates a false negative (FN) arising from environmental influences. The vessel appears smaller due to signal attenuation or scattering phenomena, resulting in a weak echo that falls below the defined detection thresholds. While tile-based training strategies facilitate handling variations in scale, they encounter difficulties in resolving subpixel features under low signal-to-noise ratio (SNR) conditions. Figure 6b shows an additional FN attributable to wave interference, where rising sea conditions produce false echoes that obscure the vessel’s signature. As a result, the oriented bounding box (OBB) head cannot isolate the target amid the available noise. Figure 6c illustrates examples of both false positives (FP) and false negatives (FN) resulting from the hierarchical inference slicing (SAHI) method. Patch splitting disrupts feature merging, leading to mismatches in intersection over union (IoU) metrics. In this case, a wave-induced artifact is erroneously classified as FP, while a genuine target is fragmented, resulting in FN.

Figure 6d shows an example of an FN resulting from target movement, where maneuvers alter the echo morphology through variations in viewing angle. The model cannot effectively detect these dynamic signatures. Figure 6e illustrates an FP scenario where a single target is misinterpreted as two separate entities, possibly due to echo splitting caused by Doppler effects or multipath reflections during maneuvers. Non-maximum suppression (NMS) is insufficient in managing such non-uniform motion patterns. Figure 6f illustrates an FN resulting from overlapping targets, where the echoes of two ships merge into a single response. Consequently, the OBB header underestimates the count; this is particularly common in areas with heavy maritime traffic.

## 4. Discussion

The results of this study show that combining tiled training with SAHI and the recently released YOLOv11-OBB architecture yields state-of-the-art performance for high-precision object detection on real-world marine radar imagery. By achieving a mean mAP@0.5 exceeding 0.95 and sub-10-pixel mean center localization errors on the challenging DAAN dataset, the proposed pipeline outperforms conventional full-image approaches, particularly in detecting small targets. These gains are especially valuable in maritime environments, where traditional axis-aligned bounding boxes often fail to capture the natural orientation of vessel wakes and asymmetric target reflections, leading to inflated bounding areas and increased false-positive rates in cluttered scenes. While color thresholding suffices for annotation in high-SNR frames, the proposed deep learning pipeline with OBB and segmentation heads provides the necessary robustness and geometric precision required for real-world maritime applications.

Compared to prior work on marine radar target detection, which has predominantly relied on classical signal-processing techniques or standard YOLO variants without tiling or slicing strategies, the current approach addresses two critical limitations simultaneously: scale variance and orientation sensitivity. Tiled training and SAHI effectively mitigate the resolution mismatch between the PPI images and the typical 640 × 640 input size of modern detectors, while the OBB head provides geometrically meaningful bounding boxes that align with the physical extent and heading of vessels.

The segmentation variant offers additional advantages for tasks such as wake suppression, target classification, or instance-level tracking, as the precise masks allow more robust separation of overlapping echoes. From a practical perspective, the lightweight YOLOv11n models (∼6 MB) combined with moderate SAHI times (4–6 FPS on an NVIDIA GeForce RTX 3050 6 GB laptop GPU) make the proposed pipeline viable for near-real-time deployment on resource-constrained systems. The trade-off is favorable for applications requiring high accuracy over maximum throughput, such as autonomous collision avoidance, small-vessel situational awareness, or integration with AIS-based tracking systems.

While the current 4–6 FPS is adequate for typical surface vessel scenarios with moderate speeds, SAHI’s slicing approach becomes particularly valuable in situations where localization accuracy and precise bounding are more critical than maximum frame rate. In such accuracy-demanding cases (e.g., dense multi-target environments, cluttered coastal waters, or close-range encounters), higher-precision detection and reduced boundary errors outweigh the slight inference slowdown introduced by SAHI.

Future work will focus on accelerating the pipeline (e.g., through model pruning, lighter backbone variants) to further improve frame rate without compromising the high mAP and sub-10-pixel localization accuracy achieved in this study. Future work will also incorporate wave-height or sea-state labels (from co-located buoy/radar measurements or hindcast models) to analyze error dependence on sea state and further validate clutter rejection in adverse conditions and incorporate cross-dataset evaluation as additional public marine radar datasets become available.

## Figures and Tables

**Table 1:** Comparison of detection performance across lightweight YOLO variants.

Model	Parameters	GFLOPs	mAP@0.5	mAP@0.5:0.95	Inference Time (ms)
YOLOv5n	2.5 M	7.1	0.981	0.787	2.7
YOLOv8n	3.0 M	8.1	0.987	0.797	2.7
YOLO11n	2.6 M	6.3	0.984	0.816	2.9
YOLO26n	2.4 M	5.2	0.987	0.807	2.8

**Table 2:** Comparison of detection performance across YOLOv11 variants and training strategies.

Model	Training	mAP@0.5	mAP@0.5:0.95
YOLOv11-detect	Resized	0.921	0.681
YOLOv11-detect	Tiled	0.984	0.816
YOLOv11-obb	Resized	0.955	0.799
YOLOv11-obb	Tiled	0.989	0.882
YOLOv11-seg	Resized	0.922	0.668
YOLOv11-seg	Tiled	0.985	0.810

**Table 3:** Effect of SAHI overlap ratio on detection performance for the YOLOv11-detect model.

Overlap Ratio	mAP@0.5	mAP@0.5:0.95	mAP@0.75
0.1	0.9619	0.4867	0.3803
0.2	0.9623	0.4877	0.3811
0.4	0.9613	0.4888	0.3917
0.5	0.9616	0.4896	0.3941

**Table 4:** Effect of SAHI slice size on detection performance for the YOLOv11-detect model with fixed overlap ratio 0.2.

Slice Size	mAP@0.5	mAP@0.5:0.95	mAP@0.75
640 px	0.9623	0.4877	0.3811
512 px	0.9729	0.5058	0.4258
448 px	0.9707	0.5085	0.4466
320 px	0.9768	0.5120	0.4860
240 px	0.9762	0.5009	0.4592

**Table 5:** Comparison of mean Average Precision (mAP) metrics across YOLOv11 variants at different IoU thresholds for SAHI.

Model	mAP@0.5	mAP@0.5:0.95	mAP@0.75
YOLOv11-detect	0.9623	0.4877	0.3811
YOLOv11-obb	0.9539	0.3743	0.4281
YOLOv11-seg	0.9476	0.4937	0.4411

**Table 6:** Precision and Recall metrics for YOLOv11 variants using tiled training and SAHI at IoU = 0.5.

Model	TP	FP	FN	Precision	Recall
YOLOv11-detect	4268	326	189	0.9290	0.9576
YOLOv11-obb	4254	409	203	0.9123	0.9545
YOLOv11-seg	4256	620	201	0.8728	0.9549

**Table 7:** Localization errors of centroids for detected vessels using the proposed tiled YOLOv11 + SAHI pipeline. Mean, median, minimum, maximum, and standard deviation of Euclidean distance (in pixels) between predicted center points and AIS-based ground-truth centers are reported separately for Vessel 2 and Vessel 4 across the three model variants.

Vessel	Model	Mean (px)	Median (px)	Min (px)	Max (px)	Std. Dev. (px)
Vessel 2	Detect	6.33	6.05	0.90	14.20	3.10
Vessel 2	Obb	6.30	6.00	1.20	14.30	3.04
Vessel 2	Segment	6.75	6.30	1.00	12.80	2.98
Vessel 4	Detect	5.14	5.20	0.10	19.00	2.99
Vessel 4	Obb	5.06	5.10	0.20	19.10	3.10
Vessel 4	Segment	5.62	5.45	0.50	20.00	2.93

## Data Availability

The dataset used in this study is available at https://doi.org/10.26090/rwmr (accessed on 26 December 2025) under DLR’s export control principles. Generated script files are available from the corresponding author on reasonable requests.

## Abbreviations

The following abbreviations are used in this manuscript:

AABB	Axis-Aligned Bounding Box
AIS	Automatic Identification System
CFAR	Constant False Alarm Rate
COCO	Common Objects in Context (dataset)
CNN	Convolutional Neural Network
FPS	Frames Per Second
GAN	Generative Adversarial Network
IoU	Intersection over Union
mAP	Mean Average Precision
NMS	Non-Maximum Suppression
OBB	Oriented Bounding Box
PPI	Plan Position Indicator
ROI	Region of Interest
SAHI	Slicing Aided Hyper Inference
SCS-GAN	Sea Clutter Suppression GAN
YOLO	You Only Look Once
